# Naringin and Naringenin Functionalized Silver Nanoparticles: Synthesis, Characterization and Biological Evaluation

**DOI:** 10.3390/pharmaceutics17121569

**Published:** 2025-12-05

**Authors:** Ozana-Andreea Măriuț, Cornelia Mircea, Bianca Ivănescu, Irina Macovei, Adrian Fifere, Irina Roșca, Ioana-Andreea Turin-Moleavin, Ana Flavia Burlec, Monica Hăncianu, Andreia Corciovă

**Affiliations:** 1 Faculty of Pharmacy, Grigore T. Popa University of Medicine and Pharmacy, 16 University Street, 700115 Iasi, Romania; stoleruozana@gmail.com (O.-A.M.); ana-flavia.l.burlec@umfiasi.ro (A.F.B.); monica.hancianu@umfiasi.ro (M.H.); maria.corciova@umfiasi.ro (A.C.); 2 Centre of Advanced Research in Bionanoconjugates and Biopolymers Department, Petru Poni Institute of Macromolecular Chemistry, 41A Grigore Ghica Voda Alley, 700487 Iasi, Romania; cornelia.mircea@umfiasi.ro (C.M.); bianca.ivanescu@umfiasi.ro (B.I.); fifere@icmpp.ro (A.F.); moleavin.ioana@icmpp.ro (I.-A.T.-M.)

**Keywords:** silver nanoparticles, naringin, naringenin, optimization, physicochemical characterization, antioxidant, antimicrobial, cytogenetic assay

## Abstract

**Background/Objectives**: Flavonoids have been extensively investigated as reducing and stabilizing agents in the green synthesis of metallic nanoparticles. However, studies specifically employing pure naringin (NG) and naringenin (NGN) remain relatively scarce. **Methods**: In the present work, silver nanoparticles (AgNPs) were synthesized under controlled laboratory conditions using NG and NGN as bioreductants, and critical parameters governing nanoparticle formation were optimized. The synthesized AgNPs were comprehensively characterized using ultraviolet–visible (UV–Vis) spectroscopy, dynamic light scattering (DLS), scanning transmission electron microscopy (STEM), energy-dispersive X-ray spectroscopy (EDX), and Fourier-transform infrared spectroscopy (FTIR). **Results**: The characterization analyses confirmed the successful formation of predominantly spherical AgNPs with average particle sizes of 17 nm (AgNG) and 20.4 nm (AgNGN). DLS analysis indicated zeta potentials of approximately −30 mV and PDIs of 0.45 (AgNG) and 0.29 (AgNGN), consistent with stable colloidal dispersions. Biological evaluations revealed that both AgNP systems exhibited notable antioxidant and antimicrobial activities. Furthermore, cytogenetic assessment using the *Allium cepa* assay demonstrated concentration-dependent alterations in mitotic index and chromosomal integrity, indicating biological activity at cellular level. **Conclusions**: Collectively, these results underscore the potential of flavonoid-mediated synthesis as an eco-friendly and effective approach for generating stable, bioactive nanomaterials with promising biological applications.

## 1. Introduction

Silver nanoparticles (AgNPs) have attracted significant attention due to their broad biological potential, including antimicrobial, anticancer, anti-inflammatory and wound-healing activity [1,2,3]. Their biological functions, together with their lower cost compared with gold or platinum nanoparticles, make AgNPs attractive for both industrial and biological applications [4].

The physicochemical properties of AgNPs, such as size, shape, surface charge and stability are closely linked to the synthesis method. Conventional physical and chemical methods often require high temperatures, toxic chemicals and expensive equipment, raising concerns about cost, safety and environmental sustainability [5]. As a safer and eco-friendly alternative, green synthesis has emerged, relying on plant-derived biomolecules as natural reducing and stabilizing agents. Plant-mediated synthesis, in particular, has gained importance due to its simplicity and abundance of phytochemicals, especially polyphenols and flavonoids, that actively reduce and stabilize silver ions during nanoparticle formation [3].

However, when using plant extracts, it is difficult to identify which specific compounds are responsible for reduction and stabilization [6]. In this context, the use of pure flavonoids, such as naringin (NG) and naringenin (NGN), allows for a more direct assessment of their role as both reducing and capping agents during nanoparticle formation.

NG (4′,5,7-trihydroxyflavanone-7-rhamnoglucoside) and its aglycone form, NGN (5,7,4′-trihydroxyflavanone), are flavanones predominantly found in citrus fruits such as grapefruit, orange, lemon and sour orange, where they contribute to the characteristic bitter taste [7,8]. Structurally, NG consists of a flavanone backbone bound to a disaccharide moiety at position C7, while NGN represents the deglycosylated form, produced in the gut through microbial hydrolysis [9]. Flavanones contain a chiral center at C2 and lack a C2=C3 double bond, which distinguishes them from other flavonoid subtypes [7].

Both NG and NGN have been studied for their antioxidant, anti-inflammatory, antitumoral, antiviral, antidiabetic and neuroprotective effects [10]. Their antioxidant activity is largely attributed to the configuration of hydroxyl groups, which enables free radical scavenger and chelate metal ions properties [8,11]. NGN, for example, can protect neuronal cells by modulating oxidative stress and inflammatory pathways, suggesting potential therapeutic relevance in disorders such as Parkinson’s and Alzheimer’s disease [10,12].

These flavonoids have also shown immunomodulatory effects. NGN can modulate PI3K/Akt and MAPK pathways, inhibit pro-inflammatory mediators including TNF-α and COX-2 and activate Nrf2 signaling, which promotes cellular defense systems [13]. Similarly, NG has demonstrated antioxidant and anti-inflammatory effects in cardiovascular, hepatic, renal and nervous tissues [14]. In addition, NG has been reported to improve bioavailability and reduce adverse effects, supporting its potential use in combination therapies [9].

Several flavonoids, including quercetin, resveratrol, curcumin and hesperidin, have been used in the green synthesis of AgNPs, yet comparatively fewer studies have examined the use of NG and NGN. Despite structural similarities with quercetin, NGN lacks the C2=C3 double bond and the 3′-hydroxyl group, features that may influence its reducing power and stabilizing capacity [15].

A review of the existing literature reveals that only a limited number of studies have explored the use of pure NG or NGN in the synthesis of AgNPs. For example, AgNPs have been obtained using aqueous solutions of pure hesperidin, NG and diosmin through a one-step sunlight-mediated synthesis, without performing optimization of reaction parameters. Their biological evaluation was limited to disc-diffusion antibacterial assays performed on a few bacterial strains, with no assessment of nanoparticle stability or correlation between conditions and biological outcomes [16]. Another study reported the sunlight-mediated synthesis of NGN–silver nanoconjugates, which were tested mainly for antimicrobial and anti-acanthamoebal activity [17]. Subsequent investigations on NGN-capped AgNPs focused on antifungal activity against *Candida albicans* or antioxidant, antibacterial, antidiabetic, anti-inflammatory and wound-healing properties in isolated assays, again employing one-step alkaline synthesis without systematic optimization or stability assessment [18,19]. More recently, a topical dermal gel containing NGN-mediated AgNPs displayed remarkable antifungal potency against *Candida albicans* and *Candida glabrata*, surpassing the activity of standard miconazole nitrate [20].

In contrast, the present study aimed to synthesize AgNPs using pure NG and NGN under controlled conditions, with particular attention given to optimizing the synthesis parameters. The resulting nanoparticles were comprehensively characterized by UV–Vis spectroscopy, dynamic light scattering (DLS), scanning transmission electron microscopy (STEM), energy-dispersive X-ray spectroscopy (EDX) and Fourier-transform infrared spectroscopy (FTIR). Their in vitro stability was also evaluated to assess the temporal stability and dispersion integrity of the colloidal suspensions. Furthermore, the antioxidant potential, antimicrobial activity and phytotoxic effects of the obtained AgNPs were systematically investigated to determine how each flavonoid influences the structural and functional properties of the nanoparticles. While several studies have explored NGN in AgNPs synthesis, reports on NG remain limited, highlighting the novelty of this direct comparative approach performed under identical synthesis, characterization and biological testing conditions.

## 2. Materials and Methods

### 2.1. Chemicals and Reagents

Naringin (≥95%), naringenin (≥95%) and silver nitrate (AgNO_3_) were purchased from Sigma-Merck (Darmstadt, Germany). All other reagents were of analytical grade.

### 2.2. Optimization of AgNPs Synthesis

The synthesis of AgNPs was monitored by recording the UV–Vis spectra (JascoV-530, Tokyo, Japan) of the reaction mixtures in the 200–600 nm range, under different experimental conditions [21]. Detailed optimization was carried out to determine the influence of each synthesis parameter on nanoparticle formation. Several parameters were systematically varied, including pH values (2, 6 and 10, adjusted with 0.1 M NaOH and 0.1 M HCl), AgNO_3_ concentrations (1, 3 and 5 mM), flavonoid/AgNO_3_ volume ratios (1:9, 5:5 and 9:1, *v*/*v*), reaction temperatures (20, 40 and 60 °C) and reaction times (0, 60, 120, 180 and 240 min). The optimization was performed in a stepwise manner, each variable being adjusted sequentially until optimal conditions for nanoparticle formation were achieved. For each variable, UV-Vis spectra were recorded and evaluated based on the position, intensity, and sharpness of the surface plasmon resonance (SPR) band.

Following optimization, the synthesis of AgNPs was performed separately using NG and NGN under their respective optimal conditions. The resulting colloidal dispersions were centrifuged at 8000 rpm for 30 min (Hettich Rotina 380 R centrifuge, Hettich, Tuttlingen, Germany), and washed three times with ultrapure water to eliminate residual impurities and to reach neutral pH, followed by separation of AgNPs. The obtained nanoparticles were then dried at 40 °C until constant weight and stored at 4 °C until further analysis.

### 2.3. Stability of AgNPs Functionalized with Naringin and Naringenin

The in vitro stability was evaluated by investigating the behavior of colloidal nanoparticles suspended in water at room temperature and under refrigeration over a period of 5 days. A volume of 5 mL of each freshly prepared colloidal solution was dispersed and homogenized with an equal amount of distilled water. The initial UV–Vis spectra of the samples were recorded, and subsequent measurements were taken after 5 days following appropriate dilution. During this period, the samples were kept at room temperature (20 °C) and in a refrigerator (4 °C).

### 2.4. Physicochemical Characterization of AgNPs

AgNPs formation was confirmed by UV–Vis spectrophotometry (JascoV-530, Tokyo, Japan). FTIR spectra were recorded for both flavonoids and the corresponding nanoparticles using a Bruker Vertex 70 spectrometer (Bruker, Billerica, MA, USA).

The hydrodynamic diameter and zeta potential were determined by dynamic light scattering (Delsa Nano Submicron Particle Size Analyzer, Beckman Coulter, Fullerton, CA, USA). Scanning Transmission Electron Microscopy (STEM) and Energy Dispersive X-ray Spectroscopy (EDX) analyses were carried out on a Verios G4 UC Scanning Electron Microscope (Thermo Scientific, Brno, Czech Republic). STEM observations were performed in STEM mode at 30 kV using a STEM 3+ detector, while the elemental composition was determined with the integrated EDX module equipped with an Octane Elect Super SDD detector (Gatan, Inc., Pleasanton, CA, USA). The surface morphology of the samples was examined using a Quanta 200 scanning electron microscope (FEI Company, Hillsboro, OR, USA) operated in low-vacuum mode at 20 kV with an LFD detector.

### 2.5. Phytotoxicity and Cytogenetic on Allium cepa

The *Allium cepa* assay was selected as an established, sensitive, and internationally recognized preliminary bioassay for assessing cytogenetic and mitotic alterations in eukaryotic systems. It provides a reliable, low-cost, and ethically non-restrictive platform suitable for screening nanoparticle-induced genotoxicity [22].

The cytogenotoxic effects of NG, NGN and their corresponding nanoparticles were evaluated by analyzing the mitotic index (MI), the distribution of mitotic phases (prophase, metaphase, anaphase, telophase), and the frequency of chromosomal aberrations induced in the meristematic cells of *Allium cepa* roots, as previously described [23]. Onion bulbs (*Allium cepa* L.) of uniform size were obtained from a local market in Iași, Romania.

The freshly emerged roots of onion bulbs were immersed in an equal volume of each sample and control (water) for 48 h. Radicles of 1.5–2 cm in length were collected post-treatment and the root tips containing the meristem tissue were stained with acetocarmine and then squashed. A minimum of 3000 cells per each sample was examined at 400× magnification, using an Eclipse E400 microscope equipped with a Nikon D3200 camera (Nikon, Tokyo, Japan). The MI was calculated as the percentage of cells undergoing division of the total number of cells counted. The percentage ratios of the cells in prophase, metaphase, anaphase or telophase were determined based on the total number of cells in mitosis. The visible chromosomal aberrations were also recorded. All experiments were performed in triplicate and the results were expressed as mean ± standard deviation.

### 2.6. Antimicrobial Activity

The antimicrobial activity of the samples was evaluated using a viable cell count method [24]. Six microbial strains were used: *Staphylococcus aureus* (*S. aureus*) ATCC25923, *Escherichia coli* (*E. coli*) ATCC25922, *Enterococcus faecalis* (*E. faecalis*) ATCC29212, *Klebsiella pneumoniae* (*K. pneumoniae*) ATCC10031, *Candida albicans* (*C. albicans*) ATCC90028, and *Candida glabrata* (*C. glabrata*) ATCC15126. Bacterial strains were subcultured on Nutrient Agar and yeast strains on Sabouraud dextrose agar, at 37 °C. From these fresh cultures microbial suspensions were prepared in sterile nutrient broth and Sabouraud dextrose broth, respectively, and adjusted to a turbidity optically equivalent to a 0.5 McFarland standard. The samples (concentration 5 mg/mL) were placed into solution which contained 0.5 mL of the microbial suspensions in culture media and 4.5 mL 1X PBS and incubated up to 24 h in a shaker at 37 °C. A microbial control experiment was also conducted. Levofloxacin (Sigma-Aldrich, St. Louis, MO, USA) and nystatin (Acos Organics, Fair Lawn, NJ, USA) were tested under the same experimental conditions as the samples and served as controls for the antibacterial and antifungal activity, respectively. At predetermined incubation times (10, 20 and 40 min, and 1, 2, 4, 6 and 24 h), 1 µL aliquots were taken from both control and treated bacterial suspensions and plated on Plate Count Agar (PCA). After 24 h of incubation at 37 °C, the colonies were counted. Each experiment was performed in triplicate to ensure reproducibility. Following incubation, the plates were evaluated using the SCAN1200^®^ system, version 8.6.10.0 (Interscience, Saint-Nom-la-Bretèche, France). Colony counts were reported as mean ± standard deviation, and the viability of treated cells was expressed as a percentage of the corresponding control.

Statistical analysis was performed using GraphPad Prism software version 7.00 for Windows (GraphPad Software, La Jolla, CA, USA, www.graphpad.com). The analysis was carried out using two-way ANOVA with Tukey’s multiple comparisons test, and differences were considered significant at *p* < 0.05.

### 2.7. Antioxidant Activity

The antioxidant activity of NG, NGN and their derived AgNPs was assessed through four in vitro assays: ferrous ion chelation, hydroxyl radical scavenging, DPPH radical scavenging and lipoxygenase (LOX) inhibition. For all assays, a reaction mixture containing all reagents except the sample was used as the negative control, while specific reference compounds were included as positive controls.

In the ferrous ion chelation assay, Fe^2+^ forms a pink complex with ferrozine, with maximum absorbance at 562 nm; the presence of chelating agents reduces the intensity of this complex. EDTA solution was used as positive control to confirm the chelating capacity of the system. Chelating activity (%) was calculated relative to the negative control according to the equation:

Chelating % = 100 × (A_c_ − A_p_)/(A_c_)

where A_c_ is the absorbance of the negative control (reaction mixture without sample) and A_p_ is the absorbance in the presence of the tested sample.

Hydroxyl radical scavenging activity was evaluated based on the hydroxylation of salicylic acid by radicals generated from the Fe^2+^/H_2_O_2_ system, producing a colored compound measured at 562 nm. Ascorbic acid solution was employed as the positive control in this assay. Scavenging (%) was calculated using the following formula:

Scavenging % = 100 × (A_c_ − A_p_)/(A_c_)

The DPPH radical assay monitored the decrease in absorbance at 517 nm as the violet radical was reduced to yellow by antioxidant compounds. Ascorbic acid solution was again used as positive control to validate the radical scavenging response. Scavenging (%) was calculated as follows:

Scavenging % = 100 × (A_DPPH_ − A_DPPH_)/(Ac)

where A_c_ is the absorbance of the DPPH solution (control) and A_p_ is the absorbance of the solution containing the tested sample.

LOX inhibition was determined by recording the reduction in absorbance at 234 nm, reflecting inhibition of linoleic acid oxidation, with ascorbic acid solution serving as the positive control for enzyme inhibition. The inhibition (%) was calculated using the following equation:

Inhibition % = (A_EFI_ − A_ECI_) × 100/A_EFI_

where A_EFI_ represents the difference between the absorbance of the enzyme solution without inhibitor at 90 s and at 30 s, and A_ECI_ represents the difference between the absorbance of the enzyme solution treated with the tested sample at 90 s and at 30 s.

Antioxidant activity (%) was calculated relative to controls. For samples with activity exceeding 50%, the effective concentration (EC_50_) values were determined by linear interpolation between the closest data points below and above 50%.

All experiments were carried out in triplicate, and results are presented as mean ± standard deviation [25]. One-way Anova was used to confirm significant differences between NG, NGN and their corresponding AgNPs.

## 3. Results

### 3.1. Synthesis Optimization of AgNPs

The synthesis of AgNPs obtained using NG (AgNG) and NGN (AgNGN) was optimized by assessing the effects of pH, AgNO_3_ concentration (1, 3 and 5 mM), flavonoid/AgNO_3_ volume ratio, temperature and stirring time. UV–Vis spectroscopy (200–600 nm) confirmed nanoparticle formation through the appearance of SPR bands within 400–450 nm [26]. A visible color shift from pale yellow to grayish-brown also confirmed reduction, with the NG system reacting more rapidly than NGN.

#### 3.1.1. pH

**Figure 1:**
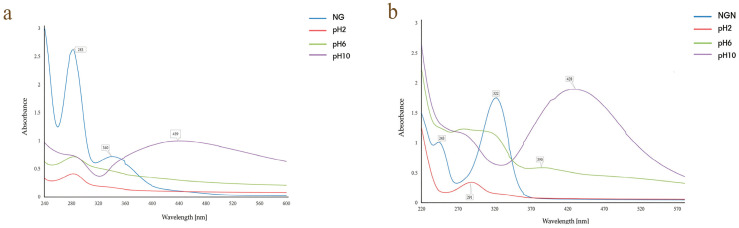
Effect of pH on the synthesis of AgNG (**a**) compared to AgNGN (**b**).

At pH 2 and 6, no distinct AgNPs bands were observed; instead, only the flavonoid absorbance signals (340 nm for NG; 322 and 243 nm for NGN) were detected, indicating inefficient reduction. In contrast, alkaline conditions (pH 10) produced SPR peaks at 439 nm (NG) and 428 nm (NGN), confirming that a basic medium favors nanoparticle formation (Figure 1a,b). Thus, pH 10 was selected for further optimization.

#### 3.1.2. AgNO_3_ Concentration

**Figure 2:**
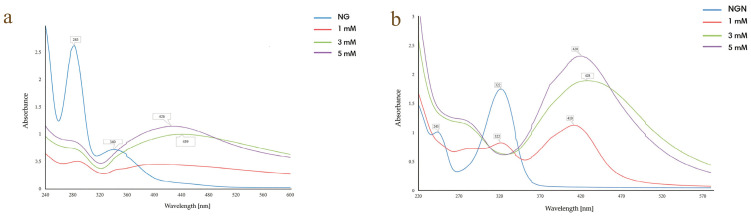
Effect of AgNO_3_ concentration on the synthesis of AgNG (**a**) compared to AgNGN (**b**).

For NG, 3 mM AgNO_3_ yielded a defined SPR band at 439 nm, which shifted to 426 nm at 5 mM, suggesting smaller particle size (Figure 2a). In the NGN system, 1 mM resulted in only a weak signal, while 3 mM and 5 mM generated stronger peaks (420 and 428 nm, respectively), with complete loss of flavonoid absorbance (Figure 2b). The optimal concentrations were set as 5 mM for NG and 3 mM for NGN.

#### 3.1.3. Flavonoid/AgNO_3_ Volume Ratio

**Figure 3:**
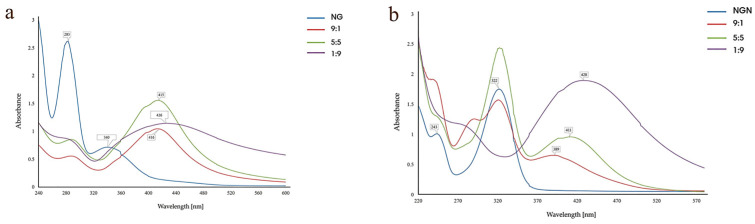
Effect of flavonoid/AgNO_3_ volume ratio on the synthesis of AgNG (**a**) compared to AgNGN (**b**).

Among the tested flavonoid/AgNO_3_ volume ratios (1:9, 5:5, 9:1), only the 1:9 ratio produced well-defined SPR bands (426 nm for NG and 428 nm for NGN), accompanied by the disappearance of the characteristic flavonoid peaks (Figure 3a,b). Excess flavonoid led to overlapping spectra and residual peaks of unreacted compounds, so the 1:9 ratio was selected for both systems.

#### 3.1.4. Temperature

**Figure 4:**
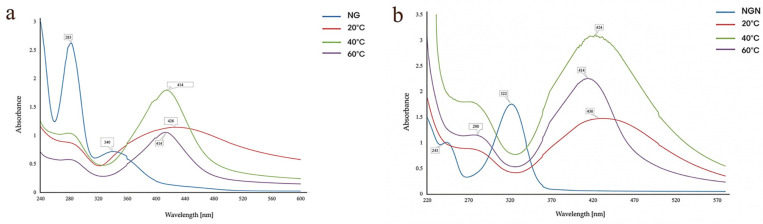
Effect of temperature on the synthesis of AgNG (**a**) compared to AgNGN (**b**).

For NG, maximum SPR intensity was obtained at 40 °C (414 nm), whereas a higher temperature (60 °C) reduced the peak intensity, suggesting heterogeneous particle sizes and aggregation (Figure 4a). At all three tested temperatures, the absorption peaks at 322 nm and 243 nm disappeared. At 40 °C and 60 °C, the absorbance intensity was higher than that observed at 20 °C; however, additional spectral signals appeared around 280 nm, which were absent at 20 °C. Therefore, further analyses will be conducted at 20 °C (Figure 4b). Accordingly, 40 °C was chosen for NG and 20 °C for NGN.

#### 3.1.5. Reaction Time

**Figure 5:**
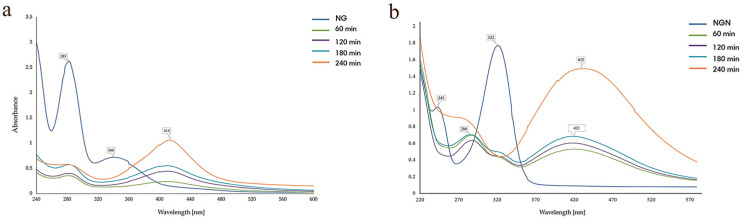
Effect of stirring time on the synthesis of AgNG (**a**) compared to AgNGN (**b**).

Regarding reaction time, for NG, the SPR band (428 nm) appeared after 60 min and intensified progressively until 240 min (Figure 5a). For NGN, after 60–180 min, the peak at 322 nm decreased in intensity and a new band emerged at 421 nm, which further shifted to 432 nm with increased intensity after 240 min, indicating complete nanoparticle formation (Figure 5b). Thus, 240 min was chosen as the optimal time for both systems.

Based on the optimization experiments, the synthesis of AgNG was the most efficient at pH 10, 5 mM AgNO_3_, a flavonoid/AgNO_3_ ratio of 1:9 (*v*/*v*), a temperature of 40 °C and a 240 min reaction time. For AgNGN, the optimal conditions were pH 10, 3 mM AgNO_3_, a flavonoid/AgNO_3_ volume ratio of 1:9, a temperature of 20 °C and a 240 min reaction time.

### 3.2. Stability of AgNPs Functionalized with Naringin and Naringenin

**Figure 6:**
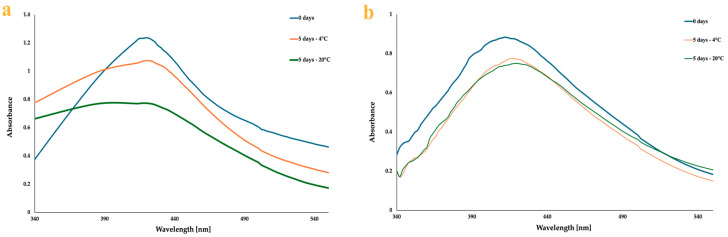
UV–Vis spectra illustrating the stability of AgNG (**a**) and AgNGN (**b**) after 5 days of storage at 20 °C and 4 °C.

In both cases (AgNG and AgNGN), a slight bathochromic shift of the absorption peak accompanied by a decrease in intensity was observed after 5 days. For AgNGN, the decrease in absorbance was approximately the same at both room temperature and refrigerated conditions, with no significant changes in peak shape (Figure 6b). In contrast, for AgNG, the peak profile broadened after 5 days and the decrease in intensity was more pronounced at room temperature (Figure 6a).

### 3.3. Physicochemical Characterization of AgNG and AgNGN

#### 3.3.1. FTIR Analysis

FTIR spectroscopy was used to identify the functional groups of NG and NGN involved in the reduction and stabilization of AgNPs.

**Figure 7:**
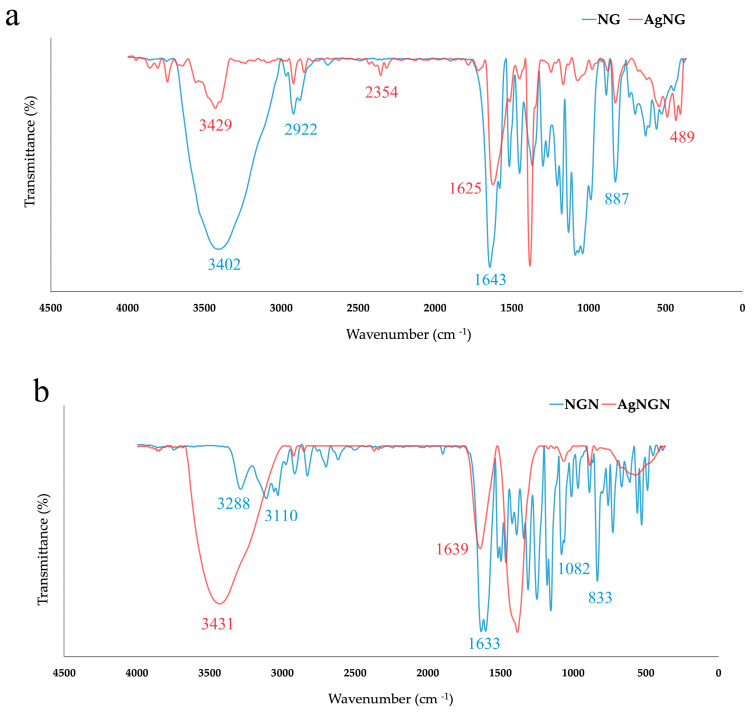
FTIR spectra of NG and AgNG (**a**) and NGN and AgNGN (**b**).

For pure NG, characteristic bands were observed at 3402 cm^−1^ (O–H stretching of alcoholic and phenolic groups), 2968–2881 cm^−1^ (C–H stretching of aromatic and aliphatic carbons) and 1643–1517 cm^−1^ (C=O stretching at position 4 and aromatic C=C) (Figure 7a). Additional peaks at 1367–1298 cm^−1^ and 1267–1039 cm^−1^ corresponded to CH_2_ deformation and C–O stretching vibrations, while the signal at 887 cm^−1^ was attributed to glycosidic β-D-pyranoside linkages [27]. After nanoparticle formation, major spectral changes were evident: the O–H stretching band shifted to 3429 cm^−1^ with reduced intensity, while the C–H stretching band disappeared. The carbonyl peak shifted from 1643 to 1625 cm^−1^ with decreased intensity and several C–O and glycosidic peaks either weakened or disappeared. The appearance of new peaks at 489 and 405–433 cm^−1^ confirmed the coordination of flavonoid moieties with silver [27].

For NGN, characteristic absorption bands were observed at 3288 cm^−1^ (phenolic O–H stretching), 3111–3058 cm^−1^ (C–H stretching of aromatic rings) and 1633 cm^−1^ (C=O stretching) (Figure 7b). Additional peaks between 1602 and 1342 cm^−1^ corresponded to aromatic C=C and CH_2_ bending, while those at 1180–1082 cm^−1^ and 889–759 cm^−1^ were associated with C–O and C–C vibrations of the flavanone nucleus [28,29,30,31]. Upon AgNPs formation, the broad O–H band shifted to 3431 cm^−1^, while the carbonyl and aromatic peaks merged into a single band at 1639 cm^−1^. Several signals in the 1517–1463 and 1342–1249 cm^−1^ regions disappeared, along with multiple C–C and glycosidic bands below 1000 cm^−1^. These changes confirm the participation of hydroxyl and carbonyl groups in silver ion reduction and stabilization [28].

#### 3.3.2. EDX Analysis

**Figure 8:**
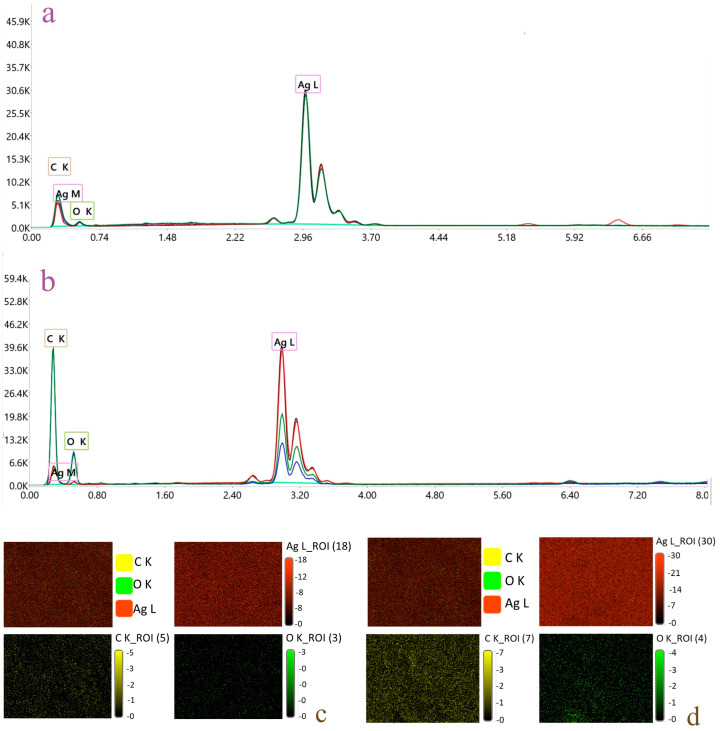
EDX analysis of AgNG (**a**) and AgNGN (**b**) in three areas; EDX elemental mapping for AgNGN (**c**) and AgNG (**d**), showing the spatial distribution of silver, carbon and oxygen.

The EDX spectra revealed a strong signal around 3 keV, confirming the presence of silver as the major component in both nanoparticle formulations. A distinct Ag peak at 3 keV is commonly reported as the elemental silver fingerprint in AgNPs and is used to confirm metallic silver rather than residual silver salts [32]. For AgNG, the composition was 82.99 wt% Ag, 10.91 wt% C and 6.09 wt% O, while AgNGN displayed a similar profile with 83.65 wt% Ag, 9.58 wt% C and 6.77 wt% O (Figure 8a,b). EDX mapping of the AgNG and AgNGN samples reveals discretely distributed, color-coded elemental patterns for Ag, C, and O at the nanoscale, consistent with the presence of nanoparticles (Figure 8c,d). In contrast, EDX mapping of the solid-phase samples shows no major discontinuities in the silver distribution, indicating that the samples remain homogeneous at the microscale. Carbon and oxygen exhibit similarly uniform elemental distributions, supporting the absence of distinct metallic or organic phases in the solid state.

#### 3.3.3. DLS Analysis

**Figure 9:**
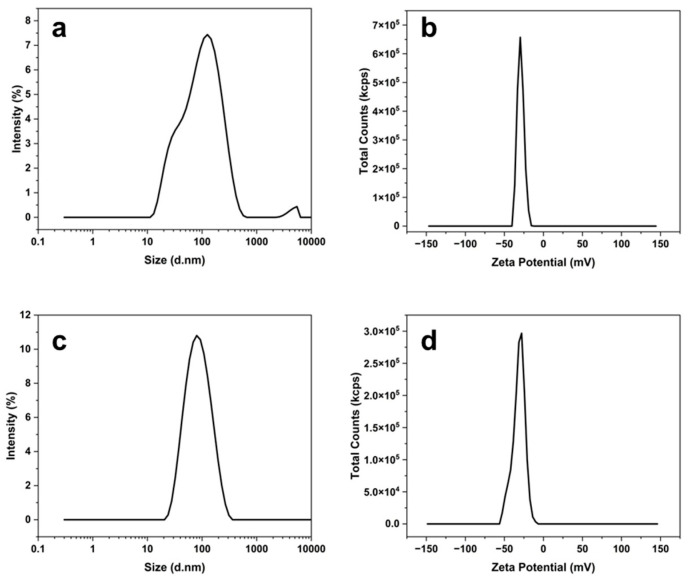
DLS analysis of AgNG and AgNGN: hydrodynamic diameter distribution (**a**,**c**) and zeta potential (**b**,**d**).

DLS analysis was used to evaluate the hydrodynamic diameter and zeta potential of the synthesized AgNPs. AgNG displayed a mean hydrodynamic diameter of 98.68 nm with a broad size distribution and a zeta potential of −29.19 mV, indicating moderate electrostatic stabilization (Figure 9a,b) [33]. Generally, nanoparticles with zeta potential values below −30 mV or above +30 mV are considered to possess high colloidal stability [34].

In comparison, AgNGN exhibited a slightly smaller average hydrodynamic diameter of 94.99 nm and a zeta potential of −31.54 mV, suggesting improved colloidal stability (Figure 9c,d). The lower polydispersity index (PDI) of AgNGN (0.29) compared to AgNG (0.45) indicates a slightly higher degree of colloidal stability in the AgNGN nanoparticles.

#### 3.3.4. STEM Analysis

**Figure 10:**
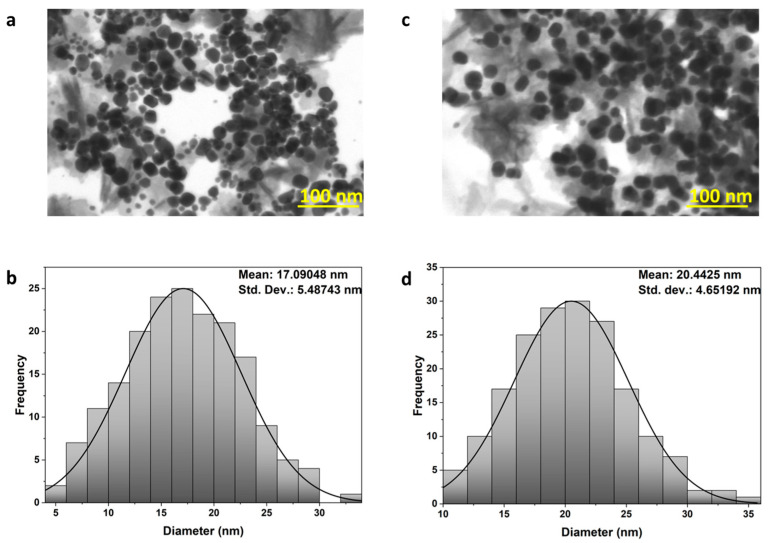
STEM micrographs of AgNG (**a**) and AgNGN (**c**) with corresponding particle size distribution histograms: AgNG (**b**) and AgNGN (**d**).

**Figure 11:**
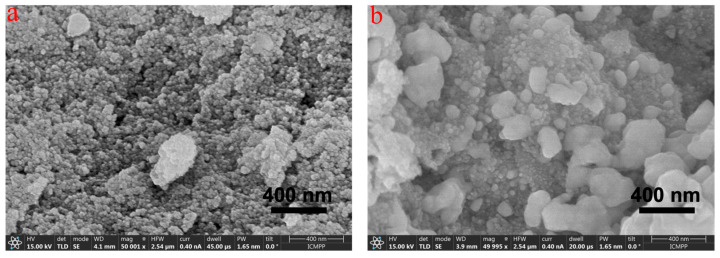
SEM micrographs of AgNGN (**a**) and AgNG (**b**).

STEM imaging revealed well-dispersed, nearly spherical nanoparticles, with no evidence of large aggregates composed of multiple particles. The average particle diameter was approximately 17 nm for AgNG and 20.4 nm for AgNGN (Figure 10), indicating a slight increase in size for the latter. SEM images reveal nanoscale structures with a predominantly spherical morphology (Figure 11a,b). These images show that AgNGN in the solid phase exhibits a slight tendency to agglomerate into globular formations, whereas in AgNG, the occurrence of larger aggregates is less pronounced.This behavior may be related to the glycosidic fragments of NG present on the AgNG surface, fragments that are absent in NGN, which can form hydrogen bonds during the drying process.

### 3.4. Phytotoxicity and Cytogenetic on Allium cepa Test

**Figure 12:**
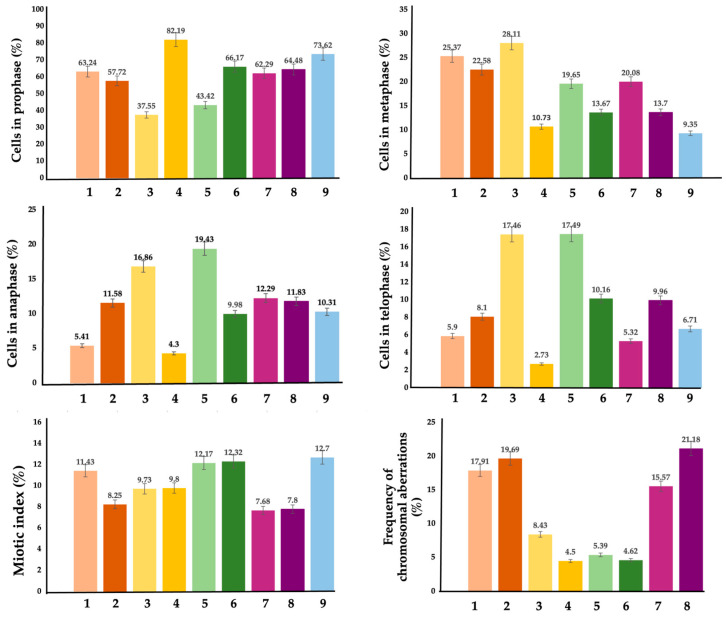
Percentages of cells in mitosis stages, mitotic index values and frequency of chromosomal aberrations in onion root meristems exposed to NG 10 mg/L (1), NG 20 mg/L (2), NGN 10 mg/L (3), NGN 20 mg/L (4), AgNG 10 mg/L (5), AgNG 20 mg/L (6), AgNGN 10 mg/L (7), AgNGN 20 mg/L (8) and water/control (9). Data are expressed as mean ± standard error (n = 3).

**Figure 13:**
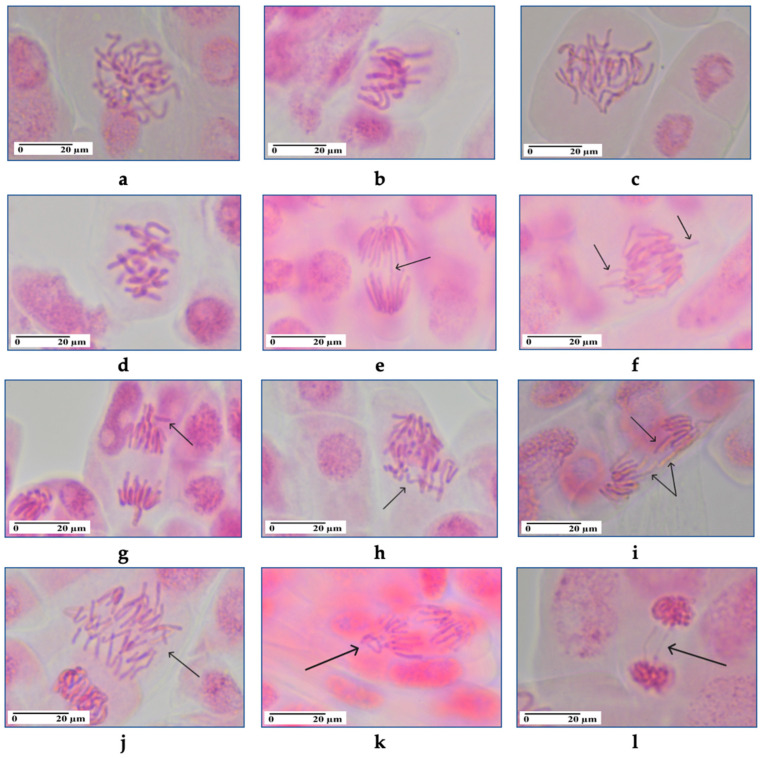
Chromosomal aberrations in onion root meristematic cells (400× magnification): (**a**). irregular prophase showing uncondensed chromosomes (AgNGN 10 mg/mL); (**b**). chromosomes with spindle disturbance at metaphase (NG 20 mg/mL); (**c**). chromosomes with spindle disturbance at early anaphase (AgNG 10 mg/mL); (**d**). C-mitosis (NG 20 mg/mL); (**e**). anaphase with chromatin bridge showed by arrow (NGN 10 mg/mL); (**f**). anaphase with microbridges and vagrant chromosomes indicated by arrows (NG 10 mg/mL); (**g**). anaphase with expulsed chromosome (arrow) and vagrants (AgNGN 20 mg/mL); (**h**). multipolar anaphase with expulsed chromosomes (arrow) (AgNGN 20 mg/mL); (**i**). disturbed anaphase with laggard chromosomes indicated by arrows (AgNGN 10 mg/mL); (**j**). monopolar anaphase (arrow) (AgNGN 10 mg/mL); (**k**). ring chromosome (arrow) at anaphase (NGN 10 mg/mL); (**l**). telophase with chromatin bridges showed by arrow (AgNGN 20 mg/mL).

Figure 12 and Figure 13 summarize the effects of NG, NGN, AgNG and AgNGN on cell division and chromosomal behavior. The results showed that AgNG at 10 mg/mL (MI = 12.17 ± 0.56%) and at 20 mg/mL (MI = 12.32 ± 0.45%) did not affect cell division, with mitotic index values similar to control (MI = 12.70 ± 0.23%). On the other hand, mitosis was inhibited by AgNGN 10 mg/mL (MI = 7.68 ± 0.33%) and 20 mg/mL (MI = 7.80 ± 0.18%) in the same way, so it appears that the genotoxicity of AgNPs was not influenced by concentration. The solution of NG 10 mg/mL slightly decreased cell division (MI = 11.43% ± 0.45%), while NG 20 mg/mL significantly inhibited mitosis (MI = 8.25 ± 0.89%). Roots treated with NGN at 10 and 20 mg/mL had a reduced number of cells in division, but similar mitotic indices (9.73 ± 0.37% and 9.80 ± 0.25%, respectively), suggesting that the antimitotic action is not concentration-dependent.

All treated samples showed chromosomal aberrations (Figure 13) such as C-mitosis, un-oriented chromosomes in disturbed metaphase, chromosomal stickiness, chromatin bridges, lost chromosomes, vagrant and laggard chromosomes at anaphase, multipolar anaphase, ring chromosomes, giant cells and polyploid prophase. The frequency of chromosomal aberrations was higher in onion root cells treated with NG and in those treated with AgNGN regardless of the concentration.

### 3.5. Antimicrobial Activity

**Figure 14:**
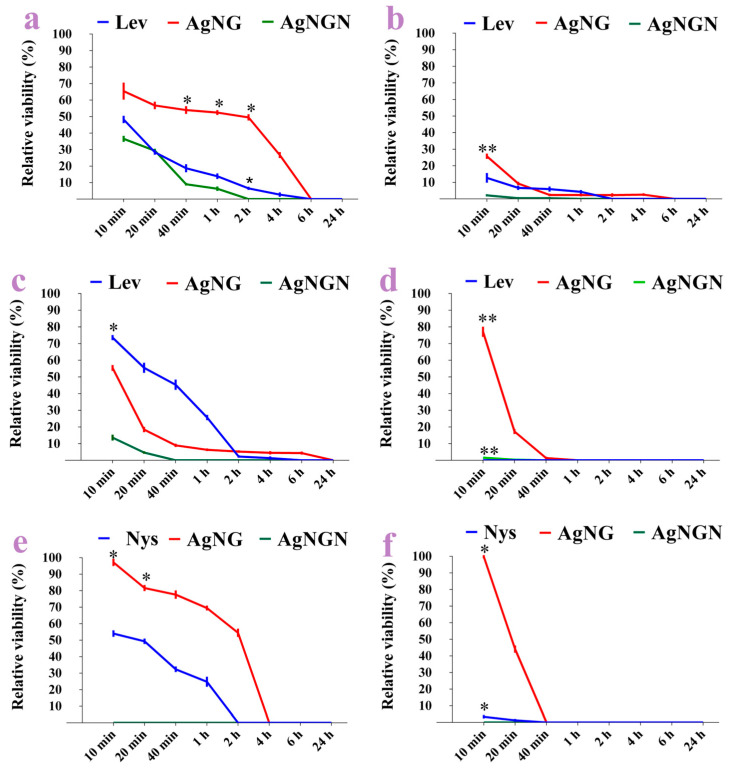
In vitro antimicrobial activity of the samples: growth inhibition of the bacterial cells ((**a**). *S. aureus*, (**b**). *E. coli*, (**c**). *E. faecalis*, (**d**). *K. pneumoniae*, (**e**). *C. albicans*, and (**f**). *C. glabrata*) expressed as percent of viable bacteria vs. incubation time (n = 3). Statistical analysis was performed using two-way ANOVA with Tukey’s multiple comparisons test. Significant differences are indicated as * *p* < 0.05 and ** *p* < 0.01. * *p* < 0.05: 10 min—Lev vs. AgNGN (*E. faecalis*), AgNG vs. AgNGN (*C. albicans*), Nys vs. AgNG and AgNG vs. AgNGN (*C. glabrata*); 20 min—AgNG vs. AgNGN (*C. albicans*); 40 min—AgNG vs. AgNGN (*S. aureus*); 2 h—Lev vs. AgNG and AgNG vs. AgNGN (*S. aureus*). ** *p* < 0.01: 10 min—Lev vs. AgNG and AgNG vs. AgNGN (*K. pneumoniae*), AgNG vs. AgNGN (*E. coli*). Abbreviations: Lev, levofloxacin; Nys, nystatin; min, minutes.

**Figure 15:**
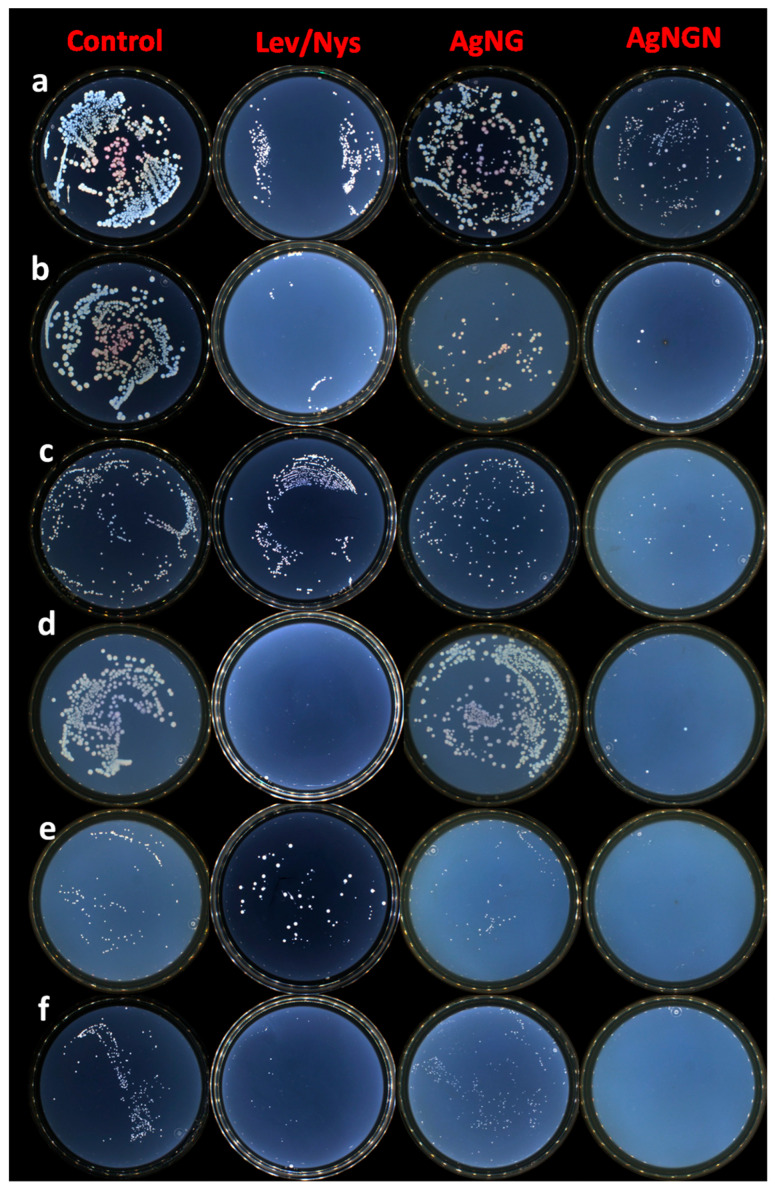
Bacterial and fungal colonies after 20 min of incubation with AgNG and AgNGN compared with microbial controls and levofloxacin/nystatin controls: (**a**). *S. aureus*, (**b**). *E. coli*, (**c**). *E. faecalis*, (**d**). *K. pneumoniae*, (**e**). *C. albicans*, and (**f**). *C. glabrata*.

Both AgNG and AgNGN displayed pronounced antimicrobial effects, whereas NG and NGN showed no activity at the tested concentrations (Figure 14 and Figure 15). All antimicrobial assays were performed using a nanoparticle concentration of 5 mg/mL, as specified in Section 2.6.

AgNGN consistently outperformed AgNG across all tested strains. Against *S. aureus*, AgNGN achieved complete inhibition within 2 h, compared to 6 h for AgNG.

In the case of *E. coli*, growth was fully suppressed after only 1 h with AgNGN, whereas AgNG required 6 h. For *E. faecalis* and *K. pneumoniae*, inhibition occurred within 40 min for AgNGN and after 1 h for AgNG. A more pronounced effect was observed against yeasts: *C. albicans* was eliminated within 10 min by AgNGN, but required 4 h for AgNG, while *C. glabrata* was eradicated in 10 min with AgNGN versus 40 min with AgNG. These results clearly demonstrate the superior and faster antimicrobial activity of AgNGN compared to AgNG.

When comparing the antibacterial activity of the samples with that of levofloxacin at the same concentration, it was observed that for *S. aureus* AgNGN exhibited greater efficiency than the antibiotic after only 2 h of incubation, whereas levofloxacin reached a comparable level of activity after 6 h and AgNG after 24 h (Figure 14a). A similar trend was observed for *E. coli*, where AgNGN exhibited significant antibacterial activity after 1 h of incubation, whereas levofloxacin showed comparable activity after 2 h, and AgNG after 6 h (Figure 14b). *E. faecalis* was also completely inactivated more rapidly by AgNGN, achieving total inhibition after only 40 min of incubation, whereas levofloxacin exhibited comparable efficacy after 6 h and AgNG after 24 h (Figure 14c). Levofloxacin proved to be more efficient than the nanoparticles in case of *K. pneumoniae*, reducing the bacterial populations to zero after only 10 min of incubation, while AgNGN had the same effect after 40 min, and AgNG after 1 h (Figure 14d).

In comparison with the antifungal control nystatin, AgNGN also demonstrated rapid activity. Against *C. albicans*, AgNGN achieved complete inactivation of fungal cells within 10 min, whereas nystatin required 2 h and AgNG 4 h to produce similar effects (Figure 14e). AgNGN was highly effective against *C. glabrata* within 10 min, while nystatin and AgNG reached similar activity after 40 min (Figure 14e). Comparable results were obtained for *C. glabrata*. AgNGN exhibited substantial activity after 10 min, while both nystatin and AgNG reached similar levels only after 40 min (Figure 14f). Statistically significant differences (* *p* < 0.05, ** *p* < 0.01) are shown for all experiments in Figure 14a–f.

### 3.6. Antioxidant Activity

**Figure 16:**
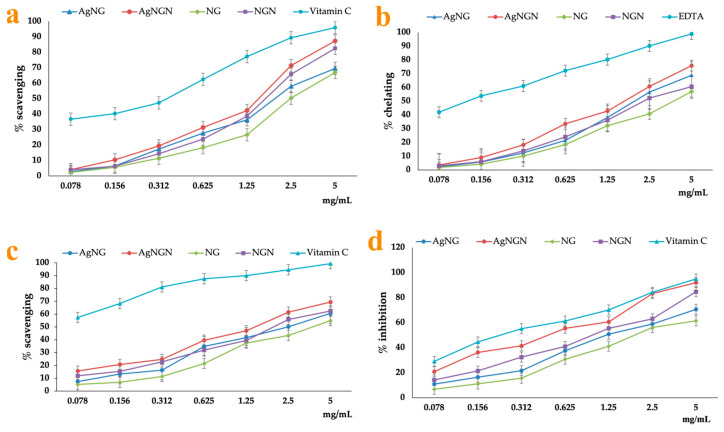
Antioxidant activity: hydroxyl radical scavenging activity (**a**), ferrous ion chelating capacity (**b**), DPPH radical scavenging activity (**c**), LOX inhibition assay (**d**). Data are expressed as mean ± standard error (n = 3). Significant differences are indicated as *p *< 0.001.

The antioxidant activity of NG, NGN and its derived AgNPs was assessed using four complementary in vitro assays: hydroxyl radical scavenging capacity, ferrous ion (Fe^2+^) chelating efficiency, DPPH radical scavenging activity and LOX inhibition (Figure 16).

In the hydroxyl radical scavenging assay, all tested samples displayed concentration-dependent activity. NGN exhibited stronger activity than NG, consistent with the higher reactivity of free phenolic hydroxyl groups, while both AgNPs formulations outperformed their corresponding flavonoids. The EC_50_ values indicated that AgNGN was the most active (140.82 µg/mL), followed by AgNG (181.47 µg/mL), NGN (156.60 µg/mL) and NG (232.04 µg/mL), confirming the superior radical-scavenging efficiency of nanoparticle systems (Figure 16a). Ascorbic acid was included as positive control in the hydroxyl radical, DPPH and LOX assays, while EDTA was used as positive control in the Fe^2+^ chelation assay.

A similar trend was observed in the ferrous ion chelation assay. Both flavonoids showed moderate chelating ability, with NGN being more active than NG. Functionalization into AgNPs further enhanced the activity, with AgNGN achieving the lowest EC_50_ value (330.18 µg/mL) compared to AgNG (390.78 µg/mL), NGN (446.54 µg/mL) and NG (740.10 µg/mL). This indicates that the AgNPs provide additional stabilization and enhance metal-binding capacity relative to the free flavonoids. EDTA exhibited the highest Fe^2+^-chelating capacity (Figure 16b).

In the DPPH radical scavenging assay, all compounds exhibited significant activity, with AgNPs consistently surpassing their free counterparts. The highest efficiency was recorded for AgNGN (EC_50_ = 143.80 µg/mL), followed by AgNG (248.54 µg/mL), NGN (195.44 µg/mL), and NG (372.94 µg/mL). Ascorbic acid displayed stronger DPPH scavenging activity than both flavonoids and their AgNPs (Figure 16c).

The lipoxygenase inhibition assay confirmed the antioxidant potential of the tested samples. Both NG and NGN were active, but their corresponding AgNPs demonstrated significantly higher inhibitory capacity. AgNGN showed the strongest effect (EC_50_ = 7.97 µg/mL), more than twice as effective as AgNG (20.02 µg/mL) and substantially higher than NGN (16.07 µg/mL) and NG (31.55 µg/mL) (Figure 16d). In this assay, the inhibitory effect of the flavonoids and their AgNPs was closer to that of ascorbic acid than in the other antioxidant tests. Similar enhancements in antioxidant activity upon nanoparticle formation have also been reported for other flavonoid-based systems, such as dihydromyricetin-stabilized AgNPs and hesperidin-based metal nanoparticles, which likewise show concentration-dependent radical scavenging and stronger antioxidant effects than the corresponding free flavonoids [35].

Overall, the results showed that both NG and NGN contribute to antioxidant activity through hydroxyl radical scavenging, metal chelation, DPPH neutralization and LOX inhibition. However, functionalization into AgNPs significantly amplified these effects, particularly for AgNGN, which consistently exhibited the highest activity across all assays, while comparison with the reference controls (ascorbic acid and EDTA) highlights the favorable antioxidant profile and improved stability of the nanoparticle formulations.

## 4. Discussion

This study is among the first to report the synthesis of AgNPs directly from NG and NGN under controlled laboratory conditions. Two previous studies relied on sunlight-assisted methods: one obtained NG-based nanoconjugates through fungal biotransformation followed by sunlight reduction, which yielded nanorods with low stability (ζ ≈ −5 mV) and reduced reproducibility [17], while another synthesized AgNPs from citrus flavonoids such as hesperidin, naringin and diosmin producing polydisperse particles (10–80 nm), with NG-derived AgNPs showing comparatively higher stability and antibacterial activity than the other two flavonoids [16].

By comparison, two recent studies demonstrated that NGN can act directly as both reducing and stabilizing agent under laboratory conditions, producing spherical AgNPs with colloidal stability and biological activities, especially antifungal and wound-healing [18,19]. Unlike the sunlight-based approaches, our method allowed systematic optimization of synthesis parameters and a direct comparison of the two flavonoids in terms of nanoparticle properties and stability.

Nanoparticle formation in both systems was confirmed by SPR bands at 400–450 nm range and the progressive decline of flavonoid-specific absorbance, indicating their dual role as reducing and capping agents [36]. Among the tested parameters, pH strongly influenced synthesis: acidic and neutral conditions (pH 2 and 6) did not favor AgNP formation, while alkaline conditions (pH 10) yielded in a clear distinct SPR peak. At pH 2 and 6, no distinct AgNP bands were observed, indicating inefficient reduction. In contrast, alkaline conditions (pH 10) produced SPR peaks at 439 nm (NG) and 428 nm (NGN), confirming that a basic medium favors nanoparticle formation in both cases [37]. This effect can be explained by the deprotonation of hydroxyl groups in alkaline medium, which enhances electron donation to Ag^+^ and thereby promotes nanoparticle nucleation and stabilization. In contrast, in acidic or neutral media, protonated –COOH or –OH groups are less reactive [38]. Thus, pH 10 was selected for further optimization. This observation is consistent with reports on myricetin and quercetin, where deprotonated hydroxyl groups enhance electron transfer to Ag^+^ [39,40].

The optimal flavonoid/metal volume ratio was 1:9, which produced well-defined SPR peaks, whereas higher flavonoid content caused spectral overlap and incomplete reduction. Similar behavior has been reported in hesperidin-based systems, where excess flavonoid reduced uniformity [41]. Temperature affected nanoparticle stability in a flavonoid-dependent manner. AgNG showed the best-defined SPR band at 40 °C, while at 60 °C, destabilization and aggregation occurred. AgNGN was most stable at 20 °C, with higher temperatures causing peak broadening and red-shifting. These findings are consistent with reports on quercetin-AgNPs and myricetin-AgNPs, where moderate heating (20–40 °C) favors stability, while higher temperatures lead to aggregation [39,40]. Reaction kinetics confirmed gradual nanoparticle formation: AgNG showed a stable SPR peak at 414 nm from 60 to 240 min, while AgNGN underwent a red shift (421 → 432 nm), indicating ongoing growth and stabilization, a trend also observed in quercetin-mediated systems [40].

Flavonoids typically exhibit two characteristic absorption bands in the UV–Vis region: band I (300–380 nm), corresponding to the cinnamoyl system of the B ring and band II (240–295 nm), associated with the benzoyl system of the A ring [42,43].

In our study, NG displayed a main absorption peak at 283 nm (band II) and a secondary peak at 340 nm (band I). Upon AgNP formation, both bands disappeared, while a new SPR peak emerged at 414 nm, confirming nanoparticle synthesis. In contrast, NGN showed a main absorption peak at 322 nm (band I) and a secondary peak at 243 nm (band II); these bands disappeared after reaction, being replaced by an SPR peak at 428 nm.

These spectral changes indicate that the hydroxyl and carbonyl functional groups of NG and NGN actively participated in the reduction of Ag^+^ ions, with the disappearance of characteristic flavonoid peaks reflecting their involvement in the complexation and stabilization of the resulting AgNPs.

In agreement with Mi et al., a plausible mechanism for AgNP formation involves the oxidation of phenolic groups through electron transfer from hydroxyl groups to Ag^+^ ions, leading to the nucleation of metallic silver (Ag^0^). The precise sequence of hydroxyl group involvement in the reduction process remains a subject of debate [44]. For NGN, Farajtabar et al. suggested that the 7-OH group is the most reactive, followed by 4′-OH, whereas 5-OH is less reactive due to intramolecular hydrogen bonding with the adjacent carbonyl group at C-4. Thus, electron transfer likely initiates at 7-OH, proceeds via 4′-OH, and only under stronger conditions involves 5-OH [45].

According to Tasca, in flavonoids such as quercetin, oxidation typically occurs at B-ring hydroxyls (3′,4′ positions), supporting the role of B-ring OH groups as key electron donors [46]. Electrochemical studies on quercetin further indicate preferential oxidation at B-ring hydroxyls (positions 3′ and 4′), with three possible tautomeric forms involving oxidation at 3,4′; 3,5,4′ or 7,3,4′. Based on these findings, an alternative mechanism can be proposed for NGN, in which oxidation may begin at the 4′-OH, followed by 7-OH and, under stronger conditions, 5-OH [47].

**Figure 17:**
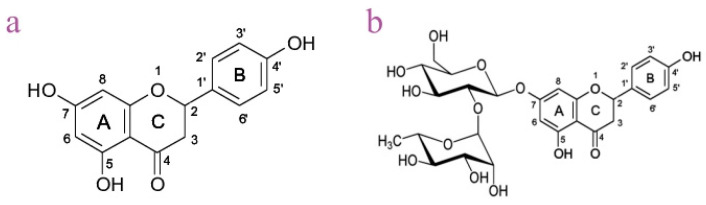
Chemical structures of naringenin (**a**) and naringin (**b**).

As illustrated in Figure 17, for NG, the 7-OH position is blocked by the rhamnoglucoside moiety, reducing its reactivity. Consequently, oxidation most likely begins at the 4′-OH group, forming a p-substituted methide quinone, with possible secondary oxidation at 5-OH under harsher conditions. However, the sequence in which the hydroxyl groups participate in the reaction remains a topic of debate.

The stability of the synthesized nanoparticles was further assessed by monitoring their UV–Vis spectra over five days under different storage conditions. Changes in the spectral profile, such as peak broadening, red shift, and a pronounced decrease in absorbance intensity, observed for AgNG after five days indicate lower stability compared with AgNGN, likely resulting from particle growth or aggregation [48,49]. According to previous reports, the stability of AgNPs is also influenced by storage conditions; in general, colloidal silver solutions stored at 5 °C in the dark exhibit the highest stability, consistent with our findings [50]. The stability of the synthesized AgNPs may also be influenced by the intrinsic properties of the capping agents [51]. In this context, NG and NGN are expected to act not only as reducing agents but also as capping molecules, forming an organic layer on the AgNP surface and providing electrostatic and/or steric hindrance that limits aggregation [52,53]. Because AgNPs tend to agglomerate due to their high surface energy, phytochemicals from plant extracts are commonly used to cover the particle surface and limit aggregation [52]. NGN has a simpler, aglycone structure, unlike NG, which is a glycosylated flavonoid and therefore more prone to degradation in aqueous media. Moreover, NGN may adsorb more efficiently onto the nanoparticle surface, forming a denser protective layer that limits aggregation. Although no previous studies have compared the stability of AgNG and AgNGN, Cordenosi et al. investigated polymeric nanoparticles loaded with these two compounds and found that NGN-loaded nanoparticles exhibited greater stability [51].

Comparative studies on flavonoid glycosides and their aglycones indicate that sugar moieties generally increase structural stability and water solubility, while aglycones show higher intrinsic activity but less stable structures. Thus, the presence of a sugar moiety in NG, in contrast to the aglycone NGN, may modulate their interaction with the nanoparticle surface and dispersion medium, with a potential impact on capping efficiency and colloidal stability [54].

**Figure 18:**
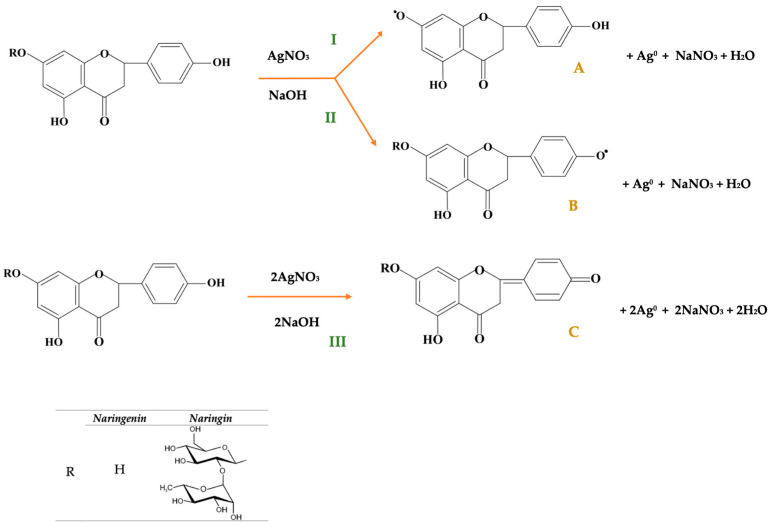
Proposed oxidation reactions of NG and NGN occurring during the formation of AgNPs.

The formation of AgNPs in the presence of NGN and NG is accompanied by the reduction of silver ions and the concomitant oxidation of the antioxidant molecules, with the probable reaction pathways illustrated in Figure 18. In reactions I and II of Figure 18, free radical intermediates are generated through the elimination of a phenolic hydrogen atom. It should be noted that, in this case, NG can participate only in reaction II, leading to the formation of the radical intermediate B. The resulting free radicals exhibit low reactivity due to electron delocalization over the aromatic rings. Nevertheless, phenoxyl radicals can be deactivated through various dimerization pathways or reactions with the solvent, which are not discussed here owing to the diversity of possible mechanisms. An alternative silver reduction pathway may also occur through reaction III, involving the oxidation of NG and NGN to yield a stable intermediate C that no longer possesses radical character.

FTIR analysis confirmed the involvement of NG and NGN functional groups in the synthesis and stabilization of AgNPs. In both systems, characteristic hydroxyl and carbonyl vibrations were evident, supporting their dual contribution to Ag^+^ reduction and nanoparticle capping. Previous studies on NGN–AgNPs have reported O–H stretching bands at 3290–3100 cm^−1^, a sharp C=O peak near 1626 cm^−1^ and additional signals corresponding to C–O (1080–1250 cm^−1^), C=C (1420 cm^−1^) and aromatic C–H (2811–3112 cm^−1^). These bands typically shift after nanoparticle formation, particularly the O–H vibration, reflecting coordination of silver ions with hydroxyl groups [19]. Comparable patterns have been observed in other flavonoid-mediated systems, such as AgNPs-Quercetin, where FTIR showed O–H stretching at 3441 cm^−1^, a carbonyl peak at 1736 cm^−1^ and characteristic C=C and C–O vibrations, highlighting the central role of phenolic and carbonyl groups in stabilization [40].

For AgNGN, the broad O–H band at 3288 cm^−1^ shifted to 3431 cm^−1^, while the C=O/C=C vibrations (1633–1602 cm^−1^) merged into a single signal at 1639 cm^−1^. Peaks in the 1517–1463 and 1342–1249 cm^−1^ regions disappeared, whereas a new band emerged at 567 cm^−1^, consistent with Ag–O bond formation [30,31]. For AgNG, similar modifications were detected: the O–H vibration at 3402 cm^−1^ shifted to 3429 cm^−1^ with reduced intensity, the C=O band at 1643 cm^−1^ shifted to 1625 cm^−1^ and several peaks in the 1517–1450 cm^−1^ and 1176–1039 cm^−1^ ranges either disappeared or shifted. Additional signals appeared at 1072 cm^−1^ and in the 433–405 cm^−1^ region, indicating Ag–O and Ag–C interactions. The disappearance of the glycosidic band at 887 cm^−1^ further suggests that the sugar moiety in NG plays a role in nanoparticle binding and stabilization [27,55].

The FTIR spectra confirmed the presence of characteristic functional groups associated with NGN and NG, supporting the conclusion that the carbon signals observed in the EDX spectra reflect, to a significant extent, the surface coverage of AgNPs by the two molecules. Both nanoparticle systems displayed a strong Ag signal, confirming the presence of metallic silver [56]. In addition, characteristic peaks of C and O were detected, consistent with the adsorption of flavonoid molecules on the nanoparticle surface. Furthermore, EDX analysis revealed Ag/C ratios of 7.6 (wt/wt) for AgNG and 8.7 (wt/wt) for AgNGN. The small difference between these values is considered negligible within the accuracy limits of EDX, suggesting that both nanoparticle types were loaded with comparable amounts of flavonoid.

DLS analysis showed hydrodynamic diameters of ~99 nm for AgNG and ~95 nm for AgNGN with zeta potentials of −29.19 mV and −31.54 mV, respectively, indicating good colloidal stability. The high negative values suggest that bioactive flavonoid molecules are adsorbed on the AgNP surface, providing electrostatic repulsion and steric hindrance that prevent agglomeration. Zeta potential thus reflects the magnitude of electrostatic interactions and is widely used as an indicator of nanoparticle stability in colloidal suspensions [57]. These values are comparable to AgNPs–Quercetin (−32.1 mV, ~100 nm) and AgNPs–Hesperidin (−28.6 mV, ~105 nm), but less negative than AgNPs–Resveratrol synthesized under alkaline conditions (−37.49 to −48.54 mV) [58]. The slightly lower PDI and slightly more negative zeta potential of AgNGN suggest marginally higher colloidal stability compared with AgNG. AgNG also showed a tendency toward bimodal DLS size distribution, whereas AgNGN remained more clearly monomodal, indicating minor aggregation in AgNG. Consistently, the higher PDI of AgNG (0.45) compared to that of AgNGN (0.29) further supports the notion that AgNG nanoparticles exhibit a slightly greater tendency for aggregation.

Electron microscopy did not reveal the presence of large, well-defined aggregates composed of nanoparticles, as both AgNG and AgNGN samples exhibited comparable morphologies and similar degrees of particle dispersion. These microscopic observations are consistent with the minor differences in aggregation tendencies inferred from zeta potential measurements and hydrodynamic diameter analyses, supporting the conclusion that both nanoparticle systems maintain comparable colloidal stability. According to international standards, the PDI reflects nanoparticle size uniformity, with lower values corresponding to more homogeneous and stable dispersions [59]. Generally, PDI values below 0.5 indicate acceptable monodispersity, while higher values suggest the presence of aggregates or broader particle size distributions [60].

STEM imaging confirmed spherical nanoparticles with average core sizes of 17 nm for AgNG and 20.4 nm for AgNGN, similar to AgNPs–Quercetin 20 nm and AgNPs–Resveratrol 11.5–17.9 nm [58]. TEM studies of AgNGN synthesized under different precursor concentrations similarly revealed spherical particles ranging from 10 to 21 nm in diameter, notably smaller than their hydrodynamic diameters determined by DLS. In contrast, DLS analysis yielded broader size distributions of 15–30 nm, with zeta potentials ranging between −25 and −33 mV, reflecting stable colloidal suspensions [16,19]. This difference between STEM/TEM and DLS was expected, because DLS measures the hydrodynamic diameter (metal core + capping layer + hydration shell), whereas STEM measures only the metallic silver core [61]. Notably, resveratrol-based metallic nanocarriers with comparable sizes have also been shown to enhance antibacterial activity relative to resveratrol alone, as the polyphenol acts both as a green reducing/capping agent and as an active antimicrobial component. Taken together, these observations suggest that the combination of small spherical core sizes, relatively high negative zeta potentials and bioactive flavonoid coatings contributes to the strong colloidal stability and enhanced antimicrobial performance of our NG–and NGN-based AgNP formulations [58].

The observed variations in hydrodynamic diameter, zeta potential and PDI between AgNG and AgNGN may contribute to their distinct biological behaviors. The slightly smaller, more uniform and more negatively charged AgNGN nanoparticles exhibited enhanced antimicrobial and antioxidant performance compared to AgNG. These results indicate that subtle physicochemical differences, particularly in surface charge and capping uniformity, can modulate nanoparticle–cell interactions, supporting the observed differences in biological efficacy [34,57].

Treatment with AgNG and AgNGN reduced the number of cells in prophase and increased the number of cells in metaphase, indicating that the cells are being arrested in metaphase. This suggests that cells were unable to progress beyond metaphase due to disruption of the mitotic spindle or interference with chromosome segregation, which also leads to chromosomal abnormalities. These findings suggest that AgNG and AgNGN act as mitotic spindle inhibitors that hamper the formation or function of microtubules which are essential for pulling chromosomes apart during cell division. The hypothesis is also sustained by the non-clastogenic chromosomal aberration identified in the meristematic cells treated with AgNPs, such as C-mitosis, vagrant and laggard chromosomes, multipolarity, chromosome stickiness, polyploidy and giant cells. Some clastogenic chromosomal aberrations were also observed in treated samples (anaphase and telophase bridges, ring chromosomes) pointing to direct DNA damage or genotoxic stress induced by treatment with AgNPs. The high frequency of chromosomal aberrations in meristematic cells treated with AgNGN correlates with the low mitotic indices of these samples.

Onion root cells treated with NG (10 and 20 mg/mL) or NGN 10 mg/mL exhibited the same behavior with reduced prophase index and increased metaphase index, and only treatment with NGN 20 mg/mL led to a rise in prophase cell count. This accumulation in prophase suggests that NGN (20 mg/mL) disrupts early mitotic events, delaying or blocking progression into metaphase. This effect may result from DNA damage, incomplete mitotic signaling, impaired spindle formation, or defects in chromosome condensation [62,63]. Activation of the spindle assembly checkpoint, triggered by improper chromosome attachment to spindle fibers, could further prevent the onset of anaphase. This mechanism may account for the reduced number of cells observed in anaphase and telophase following NGN (20 mg/mL) treatment.

To our knowledge, there are no studies on the effect of NG and NGN on plant cell mitosis, but NGN has been documented to inhibit the germination of *Arabidopsis thaliana* seeds in a dose-dependent manner, reduce root growth and disrupt gravitropism [64]. NGN also inhibits the growth and stimulates the lignification of soybean root. As an allelochemical, NGN within soil at average concentrations of 0.1 mM can affect germination and root growth of competing species [65].

On the contrary, numerous studies showed the cytotoxic effects of NG and NGN on animal cells, especially on different types of cancer cells where they were used alone, in combination with other agents, or in the form of the compound-loaded nanocarrier [66]. In animal and single-cell models, NG and NGN exhibit clear anti-proliferative activity, arresting the cell cycle at multiple points and triggering apoptotic or necrotic cell death through various molecular pathways [13]. Also, AgNG showed toxicity in human promyelocytic leukemia (HL-60) cells through generation of reactive oxygen species and cellular damage through membrane dysfunction, caspase activation, apoptosis, and DNA damage [67]. Similarly, AgNGN have shown cytotoxic effects in human colorectal cancer cells (HCT116 and HT-29) [68].

Like many polyphenols, NG shows a concentration-dependent effect: at lower doses, it is often cytoprotective (largely via antioxidant and stress-response pathways), while at higher doses, it becomes directly cytotoxic to tumor cells (via apoptosis, cell-cycle arrest), often with some selectivity over normal cells reported in vitro [69,70]. The antimitotic activity of NG, NGN and their nanoparticles could be exploited in medicine, taking into account the specific cell type, exposure time, concentration and formulation.

The *Allium cepa* test is simple, affordable, and sensitive, and has the ability to integrate multiple cytogenetic endpoints in vivo [22]. Mitotic index, phase distribution and structural/chromosomal aberrations are readily quantified in onion root meristems [71]. These endpoints correlate with mechanisms DNA strand breakage, spindle impairment, and cell-cycle arrest—that are conserved across plants and animals, making the assay an informative first screen for genotoxic/cytotoxic hazards [72]. Because AgNPs reach aquatic and terrestrial plants, the test also provides environmental relevance (root-exposure scenario, continuous contact, bioaccumulation potential) [73]. Exposure at the whole-organism level, encompassing intact physiological barriers and tissue-level metabolism, offers a more comprehensive approach than that provided by most cell lines [74].

On the other hand, phylogenetic and physiological divergence limits extrapolation of plant cytogenetic responses to human cellular processes. The lack of metabolic, immunological, and systemic complexity in *Allium cepa* prohibits accurate prediction of human toxicokinetic and dose–response relationships [72,74]. In addition, nanoparticle behavior is matrix-dependent, so corona formation, agglomeration, and Ag^+^ dissolution differ in plant media *versus* human biological fluids [75]. Differences between plant meristematic cells and mammalian systems—such as in cell-wall architecture, nanoparticle uptake and transport mechanisms, metabolic capacity, and DNA repair pathways—limit direct extrapolation to human health risk [67,76]. Additionally, nanomaterials may behave differently in biological fluids, where protein coronas, dissolution dynamics (Ag^+^ release), and interactions with mammalian enzymes can substantially modify their toxicity profile [77]. To strengthen translational relevance, future studies should include mammalian in vitro assays, such as cytotoxicity (MTT/ATP assays), oxidative stress and apoptosis markers, and mammalian micronucleus or comet assays, ideally in cell types relevant to expected exposure (e.g., epithelial cells, macrophages, hepatocyte-like cells). These should be complemented by nanoparticle characterization under biological conditions and, where justified, in vivo toxicity or biodistribution studies to establish realistic safety margins. Together, these steps will provide a more comprehensive risk assessment for potential biomedical applications of the NG/NGN–AgNP formulations.

Both AgNG and AgNGN demonstrated strong antimicrobial activity against Gram-positive bacteria (*S. aureus*, *E. faecalis),* Gram-negative bacteria (*E. coli*, *K. pneumoniae*), and yeast strains (*C. albicans*, *C. glabrata*). In all tested cases, AgNGN showed faster and more efficient eradication compared to AgNG, confirming its superior antimicrobial potency. Importantly, neither NG nor NGN alone exhibited measurable antimicrobial effects, indicating that the observed activity was strictly nanoparticle-dependent. In the majority of cases, AgNGN presented better activity than the drugs used as controls (levofloxacin and nystatin). These control drugs were generally more efficient than AgNG.

These results compare favorably with other flavonoid-based AgNPs. AgNPs–Resveratrol synthesized with sodium dodecyl sulfate (SDS) inhibited *S. aureus* and *E. coli* at 40 μg/mL, but other formulations required ≥80 μg/mL and *S. pneumoniae* was the most sensitive strain (Minimum inhibitory concentration = 40 μg/mL for SDS formulation) [58]. AgNPs–Myricetin inhibited *E. coli* and *S. typhimurium* at 10^−4^–10^−5^ g/L and caused membrane damage visible by SEM [39].

The stronger antimicrobial effect of AgNGN compared to AgNG can be attributed to structural differences between the two flavonoids. NGN, as an aglycone, is more lipophilic and penetrates bacterial membranes more efficiently, whereas NG, being a glycoside, is more hydrophilic and therefore less effective in this regard. Literature confirms that enhancing NG’s hydrophobicity or encapsulating it in carriers improves its antibacterial activity [78].

Mechanistic studies further show that NGN possesses stronger intrinsic antimicrobial activity than NG, mediated through multiple pathways: disruption of membrane integrity and fluidity, inhibition of the respiratory chain and fatty acid synthesis, blocking of quorum-sensing signals and efflux pumps, and suppression of biofilm formation [78,79]. By contrast, NG shows weaker activity but can act synergistically with antibiotics in biofilm inhibition [9].

The antimicrobial activity of AgNPs primarily involves the release of Ag^+^ ions, generation of reactive oxygen species (ROS), disruption of cellular membranes, inactivation of enzymes through thiol binding, and induction of DNA damage. When functionalized with NGN, these effects are amplified: the higher lipophilicity of NGN facilitates membrane penetration and enhances Ag^+^ ion delivery, resulting in a stronger synergistic effect compared to NG [80,81,82].

Synergies between Ag^+^ ions and flavonoids create enhanced antimicrobial, antioxidant, and anti-inflammatory properties by forming complexes that can exhibit superior efficacy to either component alone. This synergy is often leveraged in the development of AgNPs coated or loaded with flavonoids, where flavonoids improve the biocompatibility and stability of the AgNPs while the Ag^+^ ions provide antimicrobial activity leading to a more potent combined effect [83,84].

Consistent with previous reports on plant-derived AgNPs, where phytochemicals acted synergistically with silver to produce superior antimicrobial activity [82], our findings demonstrate that AgNGN exhibits significantly greater efficacy than AgNG against both Gram-positive and Gram-negative bacteria, as well as against yeast strains.

The antioxidant assays demonstrated a consistent trend: AgNPs functionalized with flavonoids exhibited superior activity compared to NG and NGN, with AgNGN consistently outperforming AgNG. These findings suggest that both the structural features of the flavonoids and their interaction with silver ions play crucial roles in determining antioxidant potential [75,77].

The antioxidant activity of NG, NGN, and their corresponding AgNPs depends on the number of hydrogen-donating functional groups present in their molecular structures. Both NG and NGN are capable of neutralizing free radicals by donating protons from hydroxyl groups, which stabilize the resulting radical species [85,86,87]. The antioxidant effect of AgNPs is more pronounced compared to that of NG and NGN because the nanoparticle surface is coated with NG or NGN molecules. Per unit mass, AgNPs contain a higher number of hydroxyl groups, and their small particle size further enhances the interaction with free radicals and lipoxygenase [16].

The higher activity of NGN compared to NG can be explained by its aglycone structure, which increases the availability of phenolic hydroxyl groups for hydrogen donation and radical scavenging. Glycosylation, present in NG, increases solubility but reduces the accessibility of active hydroxyl groups, leading to weaker antioxidant responses [88,89]. This is consistent with previous reports highlighting the superior antioxidant properties of aglycones relative to their glycosides [78,79,80,81,82].

When the results are interpreted in relation to the reference standards used in each assay, additional differences become evident. In the hydroxyl radical scavenging assay, both flavanones and their AgNPs showed lower activity than ascorbic acid, which readily donates protons and acts as a potent reducing agent [90]. In the Fe^2+^ chelation assay, EDTA exhibited a much stronger chelating capacity than the tested compounds, in agreement with its well-known ability to bind divalent metal ions and its use as a chelating agent in intoxications with divalent metals [91]. For DPPH radical scavenging, ascorbic acid again displayed superior activity compared with NG, NGN and their AgNPs, a behavior that can be attributed to its acidic character and its capacity to rapidly donate protons and electrons. However, despite this difference in potency, NG, NGN and especially their nanoparticle formulations present the important advantage of increased stability compared with vitamin C, which is more prone to oxidation and degradation [92].

Functionalization into AgNPs further amplified the antioxidant effects. The enhanced activity of AgNPs compared to free flavonoids is likely due to multiple synergistic mechanisms: improved electron transfer from deprotonated hydroxyl groups to reactive species, and increased surface area-to-volume ratio of nanoparticles, which facilitates radical interaction and stabilization of flavonoid molecules at the nanoparticle interface, preserving their activity [93,94]. The stronger activity of AgNGN compared to AgNG is therefore consistent with the more accessible hydroxyl groups of NGN, which enhance both silver ion reduction during synthesis and radical-scavenging interactions in antioxidant assays.

Among the tested assays, lipoxygenase inhibition particularly revealed strong activity for AgNGN, with EC_50_ values significantly lower than those of both free flavonoids and AgNG. This suggests a specific interaction between silver nanoparticles and enzyme active sites, where both phenolic hydroxyl groups and Ag^+^ ions may contribute to blocking substrate binding and catalysis [95,96]. Notably, in the LOX assay, the inhibitory capacity of the flavonoids and their AgNPs was much closer to that of ascorbic acid than in the other antioxidant tests, which may reflect a more complex interaction pattern involving enzyme binding and modulation rather than only simple radical scavenging [97]. Previous studies have shown that flavonoid hydroxyl groups, especially those in para positions, are effective in inhibiting LOX activity, while AgNPs can interact with enzymatic thiol groups, leading to enzyme inactivation [98,99].

Taken together, these results indicate that the antioxidant potential of NG and NGN is strongly influenced by structural differences, with NGN offering a clear advantage. When functionalized as AgNPs, these flavonoids not only retain but also enhance their antioxidant properties, likely through synergistic effects between the flavonoid moieties and silver ions. Similar synergistic enhancements have been reported for other flavonoid-functionalized AgNPs, including quercetin and resveratrol, where the combination of polyphenolic structures and nanosilver resulted in improved radical-scavenging and enzyme inhibition compared to either component alone [19,100,101].

Overall, this study shows that NG and NGN can act as both reducing and capping agents in the synthesis of AgNPs, yielding stable, spherical nanostructures with enhanced antimicrobial activity. The broad-spectrum antimicrobial activity and colloidal stability of AgNG and AgNGN underscore their potential as candidates for biological applications. Considering the fact that the present study represents a preliminary analysis of the biological potential of AgNG and AgNGN, future investigations should address long-term stability, cytotoxicity in mammalian cells, and in vivo efficacy to fully assess their translational potential.

## 5. Conclusions

In this study, AgNPs were synthesized using NG and NGN under optimized experimental conditions. The resulting AgNPs were mainly spherical with average sizes of 17 (AgNG) and 20.4 nm (AgNGN) as indicated by STEM. The colloidal stability of nanoparticles was evaluated by UV–Vis monitoring over five days, revealing that AgNGN exhibited higher stability compared to AgNG, which was also supported by zeta potentials and PDI.

The biological evaluation highlighted clear differences between the two formulations. AgNGN displayed stronger antioxidant activity, including more effective radical scavenging and lipoxygenase inhibition, and also showed faster and broader antimicrobial effects against bacteria and yeasts compared with AgNG. Neither NG nor NGN produced significant antimicrobial activity at equivalent concentrations, confirming that the biological activity was strictly nanoparticle-dependent.

Cytogenetic assays using the *Allium cepa* model revealed concentration-dependent alterations in mitotic index and chromosomal integrity, with AgNGN inducing more pronounced effects.

Overall, this study provides one of the first direct comparative analyses between AgNG and AgNGN, demonstrating how subtle structural differences between a glycosylated and an aglycone flavonoid can impact nanoparticle characteristics and biological performance. This comparative approach demonstrates that the aglycone structure of NGN promotes the formation of smaller, more stable and biologically more active nanoparticles than its glycosylated analog, NG. Such findings offer new insight into the structure–activity relationship of flavonoid-mediated AgNPs and may guide the rational design of future green nanomaterials with tailored properties.

Given their combined antimicrobial and antioxidant properties, AgNG and AgNGN appear to be promising candidates for wound healing applications, such as topical nanoformulations. Future studies should include in vivo evaluation, dose optimization and formulation development to better define their therapeutic potential and safety.

## Data Availability

The original contributions presented in this study are included in the article. Further inquiries can be directed to the corresponding authors.
